# An investigation of cognitive 'branching' processes in major depression

**DOI:** 10.1186/1471-244X-9-69

**Published:** 2009-11-10

**Authors:** Nicholas D Walsh, Marc L Seal, Steven CR Williams, Mitul A Mehta

**Affiliations:** 1 Centre for Neuroimaging Sciences, Institute of Psychiatry, King's College London, UK; 2 Melbourne Neuropsychiatry Centre, University of Melbourne, Victoria, Australia

## Abstract

**Background:**

Patients with depression demonstrate cognitive impairment on a wide range of cognitive tasks, particularly putative tasks of frontal lobe function. Recent models of frontal lobe function have argued that the frontal pole region is involved in cognitive branching, a process requiring holding in mind one goal while performing sub-goal processes. Evidence for this model comes from functional neuroimaging and frontal-pole lesion patients. We have utilised these new concepts to investigate the possibility that patients with depression are impaired at cognitive 'branching'.

**Methods:**

11 non-medicated patients with major depression were compared to 11 matched controls in a behavioural study on a task of cognitive 'branching'. In the version employed here, we recorded participant's performance as they learnt to perform the task. This involved participants completing a control condition, followed by a working memory condition, a dual-task condition and finally the branching condition, which integrates processes in the working memory and dual-task conditions. We also measured participants on a number of other cognitive tasks as well as mood-state before and after the branching experiment.

**Results:**

Patients took longer to learn the first condition, but performed comparably to controls after six runs of the task. Overall, reaction times decreased with repeated exposure on the task conditions in controls, with this effect attenuated in patients. Importantly, no differences were found between patients and controls on the branching condition. There was, however, a significant change in mood-state with patients increasing in positive affect and decreasing in negative affect after the experiment.

**Conclusion:**

We found no clear evidence of a fundamental impairment in anterior prefrontal 'branching processes' in patients with depression. Rather our data argue for a contextual learning impairment underlying cognitive dysfunction in this disorder. Our data suggest that MDD patients are able to perform high-level cognitive control tasks comparably to controls provided they are well trained. Future work should replicate these preliminary findings in a larger sample of MDD patients.

## Background

Patients with depression demonstrate impairment on a wide range of cognitive tasks. It is important to understand fully the nature of such cognitive impairment as it may have a role in the aetiology and maintainence of the disorder (MDD). Previous studies have shown impairments on tasks tapping a diverse range of cognitive functions such as memory, planning and executive attention [1-4]. Such cognitive impairment suggests a frontal-lobe basis as many of these functional impairments are present in patients with frontal-lobe dysfunction [5]. The most anterior region of the prefrontal cortex, comprising mostly BA10, is arguably the least studied region of prefrontal cortex [6]. In this study we tested the hypothesis that patients with depression are impaired on tasks associated with functioning of anterior prefrontal cortex (APFC).

Supporting evidence for such an impairment comes from a wide number of sources. Previous neuropsychological studies show relative impairments in MDD [1-4,7] on tasks that activate, amongst other regions, BA10, such as episodic and prospective memory [8], planning and higher executive functions [9,10]. A recent anatomical study by Petrides and Pandya [11] describing APFC connectivity with other regions, found strong connections with regions associated with emotional processing such as the anterior cingulate cortex and insula, allowing control over emotional and motivation functions. In line with this, a study in post-stroke depression has shown the more anterior the lesion the greater the degree of depression [12]. Another study found a negative correlation between metabolism in the frontal pole and the degree of negative cognitions scored using the Beck Depression Inventory [13], suggesting a potential link between APFC and the cognitive impairments in depression.

The most direct evidence for APFC regions contributing to cognitive deficits is reduced activation in BA10 on planning tasks in patients with depression compared to controls [14,15]. However performance on planning tasks is complex with some authors suggesting that planning processes can be further decomposed into more specific sub-processes [16]. The subprocesses involved in APFC function have not been well described on the Tower of London task. Although damage to the frontal lobes results in impairment on planning tasks such as the Tower of London. Lesions to other brain regions (e.g. cerebellum [17]) also cause impairment on this task, further complicating efforts to understand the neural basis of these performance deficits on neuropsychological tests.

The hypothesis that follows from this evidence is that patients with depression should demonstrate impairment on other tasks of APFC function. However it is only recently that tasks have been developed, for which impairment, on particular subcomponents, involves processes attributed to APFC dysfunction. Koechlin et al. [18] have shown that the APFC is activated by 'branching' processes. This involves holding in mind one goal whilst performing concurrent sub-goals. Supporting this initial fMRI finding, patients with APFC lesions are impaired at cognitive branching [19]. In the present study we have employed a version of the branching task that was modified to examine performance changes over time. An important feature of this task is that the 'branching condition' is only performed after successful completion of simpler conditions, so that impairment can be attributed to the 'branching process'. By modifying the task to measure how performance changes over time with repeated testing, we are able to accurately assess whether any impairments observed are the result of difficulties in dealing with novel rules or stimuli, or in difficulties in transferring training to a set of test stimuli.

## Methods

### Participants

12 depressed patients and 12 healthy control participants took part in this experiment. However, 1 participant from each group did not complete the experiment, and consequently we report results from 11 participants per group. All participants provided written informed consent in accordance with the guidelines of the Institute of Psychiatry/South London and Maudsley National Health Services (NHS) Trust Ethical Committee (04/Q0706/80). The depressed sample was recruited from a larger previously published study [20]. Inclusion criteria for patients was a current diagnosis of major depression as defined by Structured Clinical Interview for DSM-IV (SCID) and a Hamilton depression score > 15. Exclusion criteria for patients were any history of neurological trauma resulting in a loss of consciousness, current neurological disorder, current co-morbid Axis I disorder including bipolar disorder or an anxiety disorder, and a history of substance abuse within two months of study participation. Furthermore no patients were currently taking medication and had been free of medication for at least 1 month. Exclusion criteria for healthy controls were no history of psychiatric disorder, neurological disorder, or head injury resulting in a loss of consciousness, and a HRSD score < 7. Characteristics on these participants are described in table 1. Participants were matched for age, sex and verbal IQ.

**Table 1:** Demographic, clinical and neuropsychological measures.

	**Patients**	**Controls**
N completed =	11	11
Age	39.8 (6.8)	42.5 (9.0)
HRSD	22.8 (4.9)	- - - -
BDI	35.8 (8.1)**	2.9 (3.7)
DASS (Dep)	29.3 (8.0) **	0.5 (1.2)
DASS (Stress)	31.7 (6.7) **	3.7 (3.2)
DASS (Anx)	18.8 (10.4) **	2.2 (3.5)
RSQ	63.8 (9.4) **	27.3 (18.2)
WASI FIQ	109.9 (14.6)	117.7 (12.5)
WASI VIQ	112.2 (15.1)	114.3 (14.1)
WASI PIQ	104.4 (14.0) *	116.5 (11.3)
Digits Forwards	10.0 (2.4)	11.4 (2.3)
Digits Backwards	6.2 (2.1)	6.8 (2.9)
Digit Coding	9.8 (2.7)	11.8 (3.0)
Digit Copy	117.6 (19.0)	121.5 (16.8)
NART	111.1 (10.1)	111.4 (13.0)

*p < 0.05, **p < 0.01

Data expressed as mean (s.d.)

### Procedure

Following a brief telephone screening participants were invited to attend an initial assessment which included an interview using the SCID [21], and assessment using the Hamilton rating scale for depression (HRSD) [22] and the self-report measures: Beck Depression Inventory (BDI) [23], Beck Anxiety Inventory (BAI) [24], and the Depression Anxiety Stress Scale (DASS) [25]. A final measure, the Response Styles Questionnaire (RSQ) [26,27] that measures the degree of ruminations, was also administered during this session. Following this initial interview and depending on the experiment, participants were asked to attend a second session. In this next session they would complete some additional neuropsychological assessments and the branching task. These assessments were: the national adult reading test (NART) [28], a measure of pre-morbid IQ; Weschler abbreviated scale of intelligence (WASI) [29]; the Digit Symbol substitution and copy tests, measuring cognitive processing speed and motor speed respectively [30]; and the Digit Span [30], which comprises, the digit forwards; a measure of working memory span, and digit backwards; a measure of executive functions. To measure if any change in emotional state occured during the experiment, participants were given the Positive and Negative Affect Scale (PANAS) [31] a measure of current mood state. This was administered at the start and end of the branching task component of the experiment.

### Branching Task Procedure

**Figure 1:**
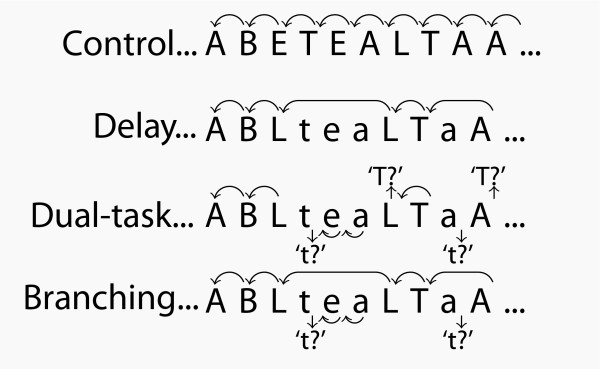
**Diagrammatic representation of rules for each condition on branching task redrawn from Koechlin et al**. [18]. In this version of the task participants completed six runs of the control condition then progressed onto the delay, dual-task and finally the branching conditions. Control condition: subjects had to decide whether two successively presented letters were also in immediate succession in the word 'tablet' (only upper-case letters were presented). Delay condition: subjects had to ignore lower-case letters which were used to occasionally delay the response required by the upper case letters. Dual task condition: subjects had to respond as in the control condition for both upper- and lower-case letter series with one exception. Subjects had to decide whether every first letter indicating a case change was the letter T (or t). Branching condition: subjects had to respond to upper-case letters exactly as in the delay condition and to lower-case letters exactly as in the dual-task condition.

This experiment used a multi-factorial paradigm which required participants to learn to predict the next letter of a sequence of letters based on a pre-specified rule (see figure 1). This experimental task was based on a study by Koechlin et al. [18] which was originally designed to assess frontal lobe cognitive control processes when a sub-goal strategy is required. The task has four conditions (control, delay, dual-task and branching) that engage additional and interacting attentional and working memory processes. The task required participants to respond to yellow letter stimuli (500 ms duration, every 3,000 ms) on a blue background displayed on a computer screen in a quiet testing room. The right or left button of a button box was to be pressed depending on whether a letter followed correctly in the sequence or not. Every participant completed 6 runs (1 run = 28 trials) of each condition in the order described below (task instructions were re-iterated to participants after every run if required).

## Results

### Demographic, clinical measures and neuropsychological measures

There results are displayed in table 1. There were no differences in age or gender between patients and controls. Patients showed significantly higher scores on the HRSD and BDI, DASS and RSQ. No patients were taking medication at the time of study. There were no differences between patients and controls on the digit span, digit symbol, NART, WASI full-scale IQ and WASI verbal IQ. Patients however scored lower on the WASI performance IQ measure (t_22 _= 2.3, p < 0.05).

### Branching task

One participant from each group did not complete the experiment. One patient only completed the first condition and one control did not complete the last condition. Therefore this data shows the performance from 11 patients and 11 controls.

**Figure 2:**
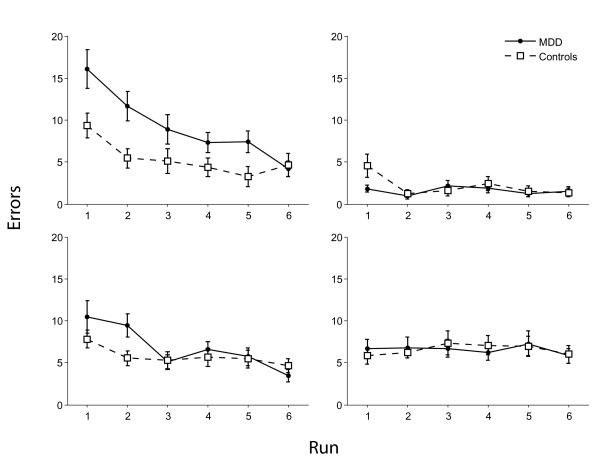
**Accuracy of controls and patients during four branching task conditions**. Top left, control condition; top right, delay condition; bottom left, dual-task condition; bottom right, branching condition.

**Figure 3:**
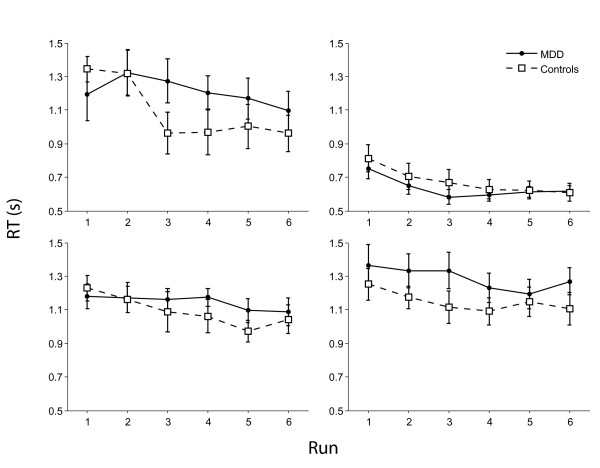
**Reaction times of controls and patients during the four branching task conditions**. Top left, control condition; top right, delay condition; bottom left, dual-task condition; bottom right, branching condition.

For performance accuracy (see figure 2) there was a main effect of condition (F_3,60 _= 27.0, p < 0.001) with all participants being more accurate on the delay condition. There was a main effect of run (F_5,95 _= 21.0, p < 0.001) with participants becoming more accurate from runs 1-6. There was a condition × run × group interaction (F_15,285 _= 2.4, p < 0.05) with patients being more impaired on runs 1 and 2 of the control condition (Fs_1,22 _= > 7.1, Ps < 0.05 but otherwise performing as well as the control group. For reaction times (see figure 3) there was a main effect of condition (F_3,57 _= 56.5, p < 0.001) with participants being faster on the delay condition but no group × condition interaction. There was a main effect of run (F_5,95 _= 12.3, p < 0.001) with participants generally responding faster with practice and a group × run interaction (F_5,95 _= 2.4, p < 0.05) explained by controls becoming faster with each run (F_5,50 _= 16.9, p < 0.001) but patients showing a small but non-significant reduction in response times(F_5,50 _= 2.14, N.S.).

#### Association of performance with symptom severity

**Figure 4:**
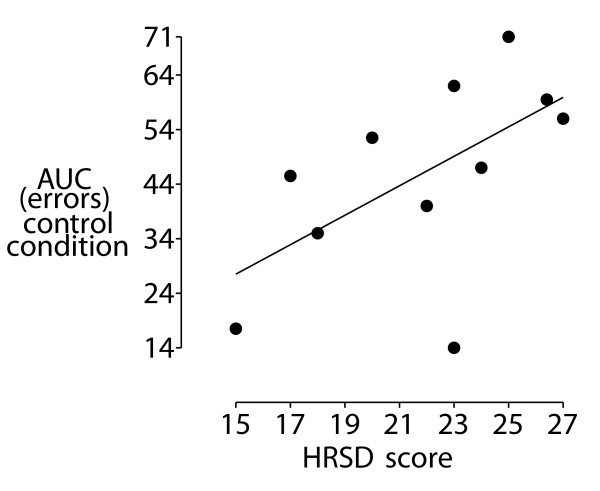
**Scatter plot of correlation of Hamilton rating scale for depression score and Area-under-curve measure of errors in performance, r = 0.63, p < 0.05**.

We wanted to establish if performance on the branching task was associated with greater illness severity in patients. To do this we performed correlations between symptom measures and two indices of behavioural performance; area-under-the curve (AUC) for error scores and reaction time. There was a significant correlation between HRSD score and AUC errors on the control condition (r = 0.63, p < 0.05, see figure 4). There was also a non-significant trend between DASS_*ANX *_score and AUC errors on the control condition (r = 0.51, p = 0.11). No other correlations were significant.

There was no association between AUC reaction time scores and symptom measures for any task condition. In exploratory analyses we found a significant positive association between DASS_*ANX *_score and reaction time on the first block of the control condition (r = 0.73, p < 0.05).

### Mood-state measures

We wanted to examine if there was a significant effect of performing the branching task on self-reported positive and negative affect. These results are displayed in table 2. We had to exclude one patient from this analysis due to missing data. For positive affect there was a main effect of group (F_1,21 _= 15.1, p < 0.001), with patients showing lower positive affect (PA) at both time points. There was also a main effect of time (F_1,21 _= 9.3, p < 0.05) which appeared to be driven by the patients. This was confirmed by a group × time interaction (F_1,21 _= 14.0, p < 0.05) with patients showing a bigger increase in PA at the end of the session. These results were mirrored by the negative affect (NA) measure also showing a main effect of group (F_1,21 _= 13.4, p < 0.001) and time (F_1,21 _= 8.1, p < 0.05). Again there was a significant group × time interaction (F_1,21 _= 12.8, p < 0.05), explained by patients showing a greater decrease in NA than controls at the end of the testing session.

**Table 2:** PANAS scores for controls and patients before and after performing the branching task.

	**Patients**	**Controls**
PA1	17.6 (4.7)	31.7 (4.7)
PA2	23.7 (6.9)	31.1 (7.0)
NA1	20.5 (9.5)	10.4 (0.5)
NA2	16.8 (6.6)	11.0 (1.8)

PA = positive affect, NA = negative affect, 1 = pre-branching task, 2 = post-branching task. Data expressed as mean (s.d.).

#### Relation between performance and affect change

There was no significant associations between any accuracy or reaction time measures on the branching task and affect change.

## Discussion

The main finding from this study is that patients with MDD were not impaired on a task measuring cognitive 'branching' processes compared to healthy controls. Due to the modified design of our paradigm we were able to examine changes in performance over time. This revealed the normal effect of reaction time shortening was attenuated in the depressed cohort. Despite the small numbers it is difficult to argue for inadequate sensitivity of the task since we were able to detect impairments in accuracy on the control condition. Taken with the reaction time attenuation this suggests a contextual learning deficit in the depressed group. Furthermore deficits have been found in cognitive branching with patients with APFC lesions (n = 7) [19]. In this study [19], the effect size found was 0.44.

Before conducting this study we conducted a power calculation based on the findings from [3]. In this study Elliott et al. [3] used a version of the Tower of London task, a test previously shown to be sensitive to APFC lesions (see introduction). From the Elliott et al. [3] report, we used the main effect of group (shown on page 982, and displayed in Figure three a in that report). The main effect of group statistic reported in this study was (F (1,48) = 22.62, p < 0.001). Using the effect sizes calculator [ (last accessed Aug 27th 2009)], we were able to calculate the effect size of 1.38 from the F value. We then used the power and sample size calculator from this web site [ (last accessed Aug 27th 2009)]. With the effect size of 1.38 from [3], we needed to study 9 experimental subjects and 9 control subjects to be able to reject the null hypothesis that the population means of the experimental and control groups are equal with probability (power) 0.8 and alpha of 0.05. Therefore we argue that we had sufficient sample size to examine our question of interest.

The reduced performance in the control task tells us that patients may need a significantly greater amount of training, and that with this, their performance can equal that of controls. Importantly, the lack of difficulty on the subsequent conditions indicates that the patients with MDD were able to deal with novel rules and transfer training to a set of test stimuli, once adequate training on the control task was given. Thus despite initial difficulties on the control condition and generalised reaction time deficits across the runs, performance accuracy on the branching condition and those leading up to it in MDD was equivalent to the control group. Whether this normal performance in branching processes is accompanied by abnormal activity or metabolism in the anterior prefrontal cortex cannot be determined by this study alone. Nonetheless it remains possible that the early learning deficits are reflective of impairments when there is greater uncertainty about what response to make in a novel situation. This occurred in the early stages of the control condition, when participants had to learn to perform the task. Such open-ended cognitive processing has also been associated with anterior prefrontal cortex function [32,33]. However we do question the reliability of deficits in complex cognitive function in previous studies, where prolonged learning phases may not have been included. This is an important line of research that should be examined in future work.

For the control condition, we found that the greatest impairment was associated with greatest illness severity. There was also evidence that the performance impairment on the control condition was associated with anxiety symptoms of the patients, as measured by the anxiety subscale of the DASS measure.

Although we excluded patients with co-morbid anxiety disorders, there could still be anxiety symptoms present, though not enough to fulfill criteria for an anxiety disorder.

We found no group differences on our other cognitive measures except for the WASI performance IQ measures. It is possible that larger samples are needed for significant effects to be shown with these other measures, again reinforcing the strength of the present findings. A criticism of the WASI performance measure is that impairment on the two subtasks could be due to a number of different cognitive processes, and lacks the precision of the branching paradigm. Importantly the WASI performance was not correlated with performance at any stage of the branching task making it difficult to attribute the changes during the control condition, or overall reaction time slowing to general ability as indexed by the WASI. Thus these control measures argue against again non-specific cognitive impairment in depression, but rather a specific impairment in learning.

An interesting finding was the change in the patients mood throughout the course of the branching experiment. They showed both an increase in positive affect and a decrease in negative affect, compared to controls whose mood was stable throughout. This may be due to a distraction effect by being absorbed in a complex task. Additionally, it is notable that a predictive version of this task activates brain regions associated with reward, namely the caudate and nucleus accumbens in healthy volunteers [10]. The activation of reward circuitry may promote change in affect, which may be baseline dependent. However, no control task, or testing session, was included in this study and therefore further study of the critical processes underlying this effect is warranted. A related issue is the role of the experimenter (NDW) in the present task, who may have provided a form of positive social support during testing. Anecdotally, a number of patients became tearful on the first few runs of the control condition, but were able to continue. It is unclear whether this was a significant factor in the mood-change, and should be investigated in further work.

It is important to emphasise that the depressed sample, who were carefully selected to be unmedicated, with a moderate degree of depression and no co-morbidities was comparable to other depressed samples in terms of the severity of the symptoms [20,34]. Therefore the lack of a deficit in the branching, dual-task and delay conditions of the task cannot easily be explained by the fact that these were an atypical high functioning group.

However there are a number of limitations in this study that are important to discuss. Firstly, although non-medicated patients participated we did have a relativly small sample size of 11 patients. This is important as depression is a heterogeneous disorder and it is conceivable that although we did not demonstrate a branching impairment in our sample there may be illness subtypes that are impaired on this task.

Secondly, it is unclear whether the impairment in the patient group on the control condition was due to impairment on performing the control condition *per se *or because this was the first experimental condition participants completed. In itself, performing this control condition involves a substantial degree of cognitive processing. Although the other conditions build on the control conditions and *are *more difficult, we believe that the cognitive operations required in the control condition are by no means simple from an information-processing perspective. Learning this condition requires the participant to hold in mind letters from the word 'tablet' and to update their memory depending on what letter occurred previously.

Therefore this updating process, of whether a target letter matches their expectation or not, and then pressing the correct button, appears to be a sensorimotor skill learning process that becomes more automatic with increasing practice (from runs 1-6). It is however unclear at present from this data which of the cognitive processes involved are the specific processes that patients with depression are impaired on. It could be a number of things, such as the ability to 'hold in mind' the letters from the word 'tablet', it could be adjusting to the pace of the task, or it could be a problem in dealing with feedback that does not match their expectation and updating their subsequent predictions of what letter will occur next. There is much work to be conducted in future experiments to clarify the nature of the impairment in the control condition. We have recently shown that these processes can be dissociated behaviourally and neurally in healthy participants [35], but it is currently unknown which of these specific processes are impaired in depressed adults.

Thirdly, a result of running the conditions sequentially we were unable to separate out the condition and order effects. It is worth noting however that in previous published studies using this task the learning of each condition is not explicitly recorded. In this study we have explicitly recorded the learning (across six blocks) and the fixed order of conditions simplifies the learning procedure considerably as each condition builds on the previous one. Now that we have shown that these conditions can be accurately learned in patients with depression, future experiments can be conducted on pre-trained participants where the conditions are administered in a counter-balanced order.

A final limitation pertaining to the mood-state measures, is that we did not have a 'waiting-list control' patient group to conclusively determine that it was the branching task that caused the improvement in the patient's mood and not another factor such as contact with the experimenter or other non-specific distraction.

## Conclusion

Our data tentatively support the hypothesis of a contextual learning deficit in patients with major depression. This study thus provides an exciting development in understanding the role of cognition in major depression. Future work is needed to further clarify the details of such impairment. Our data suggest that MDD patients are able to perform high-level cognitive control tasks comparably to controls provided they are well trained.

## Competing interests

The authors declare that they have no competing interests.

## Authors' contributions

NDW, SCRW, MLS, MAM conceived and designed the experiment. NDW conducted the experiment. NDW and MAM performed the data analysis. NDW, MLS and MAM wrote the paper. All authors have read and approved the final version of the manuscript.

## Pre-publication history

The pre-publication history for this paper can be accessed here:



## References

[B1] Weingartner H, Cohen RM, Murphy DL, Martello J, Gerdt C (1981). Cognitive processes in depression. Arch Gen Psychiatry.

[B2] Hertel PT, Hardin TS (1990). Remembering with and without awareness in a depressed mood: evidence of deficits in initiative. J Exp Psychol Gen.

[B3] Elliott R, Sahakian BJ, McKay AP, Herrod JJ, Robbins TW, Paykel ES (1996). Neuropsychological impairments in unipolar depression: the influence of perceived failure on subsequent performance. Psychol Med.

[B4] Channon S, Green PS (1999). Executive function in depression: the role of performance strategies in aiding depressed and non-depressed participants. J Neurol Neurosurg Psychiatry.

[B5] Burgess PW, Alderman N, Forbes C, Costello A, Coates LM, Dawson DR, Anderson ND, Gilbert SJ, Dumontheil I, Channon S (2006). The case for the development and use of ecologically valid measures of executive function in experimental and clinical neuropsychology. Journal of the International Neuropsychological Society.

[B6] Ramnani N, Owen A (2004). Anterior prefrontal cortex: Insights into function from anatomy and neuroimaging. Nature Reviews Neuroscience.

[B7] Rude SS, Hertel PT, Jarrold W, Covich J, Hedlund S (1999). Depression-related impairments in prospective memory. Cognition and Emotion.

[B8] Burgess PW, Scott SK, Frith CD (2003). The role of the rostral frontal cortex (area 10) in prospective memory: a lateral versus medial dissociation. Neuropsychologia.

[B9] Konishi S, Chikazoe J, Jimura K, Asari T, Miyashita Y (2005). Neural mechanism in anterior prefrontal cortex for inhibition of prolonged set interference. Proc Natl Acad Sci USA.

[B10] Koechlin E, Corrado G, Pietrini P, Grafman J (2000). Dissociating the role of the medial and lateral anterior prefrontal cortex in human planning. Proc Natl Acad Sci USA.

[B11] Petrides M, Pandya DN (2007). Efferent association aathways from the rostral prefrontal cortex in the macaque Monkey. J Neurosci.

[B12] Starkstein SE, Robinson RG, Price TR (1987). Comparison of cortical and subcortical lesions in the production of poststroke mood disorders. Brain.

[B13] Dunn RT, Kimbrell TA, Ketter TA, Frye MA, Willis MW, Luckenbaugh DA, Post R (2002). Principal components of the Beck Depression Inventory and regional cerebral metabolism in unipolar and bipolar depression. Biol Psychiatry.

[B14] Elliott R, Baker SC, Rogers RD, O'Leary DA, Paykel ES, Frith CD, Dolan RJ, Sahakian BJ (1997). Prefrontal dysfunction in depressed patients performing a complex planning task: a study using positron emission tomography. Psychol Med.

[B15] Goethals I, Audenaert K, Jacobs F, Van de WC, Ham H, Pyck H, Vandierendonckc A, Van Heeringenb C, Dierckxet R (2005). Blunted prefrontal perfusion in depressed patients performing the Tower of London task. Psychiatry Res.

[B16] Goel V, Grafman J (1995). Are the frontal lobes implicated in "planning" functions? Interpreting data from the Tower of Hanoi. Neuropsychologia.

[B17] Grafman J, Litvanm I, Massaquoi S, Stewart M, Sirigu A, Hallet M (1992). Cognitive planning deficit in patients with cerebellar atrophy. Neurology.

[B18] Koechlin E, Basso G, Pietrini P, Grafman J (1999). The role of the anterior prefrontal cortex in human cognition. Nature.

[B19] Dreher JC, Koechlin E, Tierney M, Grafman J (2008). Damage to the fronto-polar cortex is associated with impaired multitasking. PLoS ONE.

[B20] Walsh ND, Williams SC, Brammer MJ, Bullmore ET, Kim J, Suckling J, Mitterschiffthaler MT, Cleare AJ, Pich EM, Mehta MA, Fu CH (2007). A longitudinal functional magnetic resonance imaging study of verbal working memory in depression after antidepressant therapy. Biol Psychiatry.

[B21] First MB, Spitzer RL, Gibbon M, Williams JBW (1995). Structured Clinical Interview for DSM-IV Axis I Disorders.

[B22] Hamilton M (1960). A rating scale for depression. J Neurol Neurosurg Psychiatry.

[B23] Beck AT, Steer RA (1987). Manual for the revised Beck Depression Inventory.

[B24] Beck AT, Steer RA (1990). Manual for the Beck Anxiety Inventory.

[B25] Brown TA, Korotitsch W, Chorpita BF, Barlow DH (1997). Psychometric properties of the Depression Anxiety Stress Scales (DASS) in clinical samples. Behaviour Research and Therapy.

[B26] Kasch KL, Klein DN, Lara ME (2001). A construct validation study of the Response Styles Questionnaire Rumination Scale in participants with a recent-onset major depressive episode. Psychol Assess.

[B27] Nolen-Hoeksema S (1991). Responses to depression and their effects on the duration of depressive episodes. J Abnorm Psychol.

[B28] Nelson HE, Willinson J (1991). National Adult Reading Test Manual.

[B29] Wechsler D (1999). Weschler abbreviated scale of intelligence.

[B30] Wechsler D (1988). Weschler Adult Intelligence - Revised.

[B31] Watson D, Clark LA, Tellegen A (1988). Development and validation of brief measures of positive and negative affect: the PANAS scales. J Pers Soc Psychol.

[B32] Daw ND, O'doherty JP, Dayan P, Seymour B, Dolan RJ (2006). Cortical substrates for exploratory decisions in humans. Nature.

[B33] Yoshida W, Ishii S (2006). Resolution of uncertainty in prefrontal cortex. Neuron.

[B34] Fu CH, Williams SC, Cleare AJ, Brammer MJ, Walsh ND, Kim J, Andrew CM, Pich EM, Williams PM, Reed LJ, Mitterschiffthaler MT, Suckling J, Bullmore ET (2004). Attenuation of the neural response to sad faces in major depression by antidepressant treatment: a prospective, event-related functional magnetic resonance imaging study. Arch Gen Psychiatry.

[B35] Walsh ND, Phillips ML Interacting Outcome Retrieval, Anticipation, and Feedback Processes in the Human Brain. Cereb Cortex.

